# Kinetics, Products, and Brown Carbon Formation by Aqueous-Phase Reactions of Glycolaldehyde with Atmospheric Amines and Ammonium Sulfate

**DOI:** 10.1021/acs.jpca.2c02606

**Published:** 2022-08-04

**Authors:** Alyssa A. Rodriguez, Michael A. Rafla, Hannah G. Welsh, Elyse A. Pennington, Jason R. Casar, Lelia N. Hawkins, Natalie G. Jimenez, Alexia de Loera, Devoun R. Stewart, Antonio Rojas, Matthew-Khoa Tran, Peng Lin, Alexander Laskin, Paola Formenti, Mathieu Cazaunau, Edouard Pangui, Jean-François Doussin, David O. De Haan

**Affiliations:** † Department of Chemistry and Biochemistry, University of San Diego, 5998 Alcala Park, San Diego, California 92110, United States; ‡ Department of Chemistry, Harvey Mudd College, 301 Platt Boulevard, Claremont, California 91711, United States; § Environmental Molecular Sciences Laboratory, Pacific Northwest National Laboratory, Richland, Washington 99352, United States; ∥ Department of Chemistry, Purdue University, West Lafayette, Indiana 47907, United States; ⊥ Laboratoire Interuniversitaire des Systèmes Atmosphériques (LISA), UMR7583, CNRS, Université Paris-Est Créteil (UPEC) et Université de Paris, Institut Pierre Simon Laplace (IPSL), 94000 Créteil, France

## Abstract

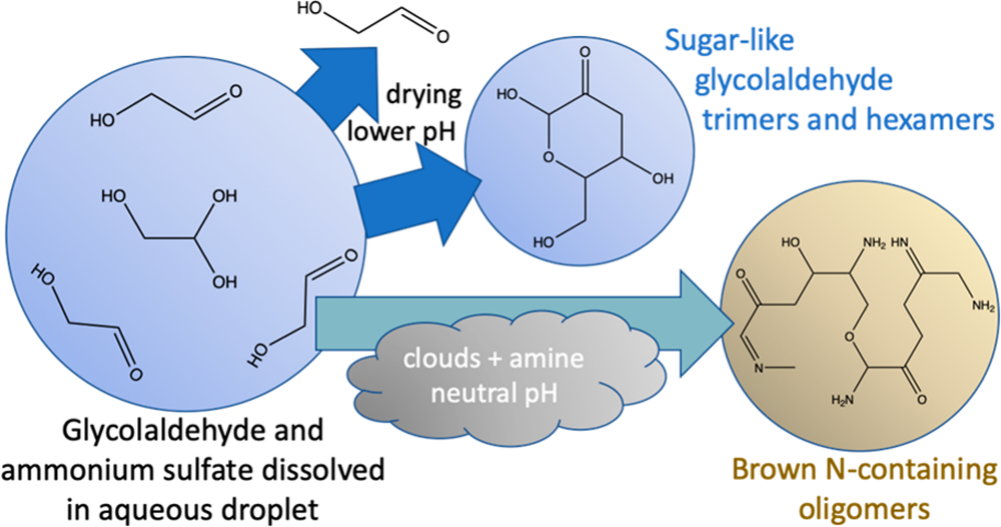

Glycolaldehyde (GAld) is a C_2_ water-soluble aldehyde produced during the atmospheric oxidation of isoprene and many other species and is commonly found in cloudwater. Previous work has established that glycolaldehyde evaporates more readily from drying aerosol droplets containing ammonium sulfate (AS) than does glyoxal, methylglyoxal, or hydroxyacetone, which implies that it does not oligomerize as quickly as these other species. Here, we report NMR measurements of glycolaldehyde’s aqueous-phase reactions with AS, methylamine, and glycine. Reaction rate constants are smaller than those of respective glyoxal and methylglyoxal reactions in the pH range of 3–6. In follow-up cloud chamber experiments, deliquesced glycine and AS seed particles were found to take up glycolaldehyde and methylamine and form brown carbon. At very high relative humidity, these changes were more than 2 orders of magnitude faster than predicted by our bulk liquid NMR kinetics measurements, suggesting that reactions involving surface-active species at crowded air–water interfaces may play an important role. The high-resolution liquid chromatography–electrospray ionization–mass spectrometric analysis of filter extracts of unprocessed AS + GAld seed particles identified sugar-like C_6_ and C_12_ GAld oligomers, including proposed product 3-deoxyglucosone, with and without modification by reactions with ammonia to diimine and imidazole forms. Chamber exposure to methylamine gas, cloud processing, and simulated sunlight increased the incorporation of both ammonia and methylamine into oligomers. Many C_4_–C_16_ imidazole derivatives were detected in an extract of chamber-exposed aerosol along with a predominance of *N*-derivatized C_6_ and C_12_ glycolaldehyde oligomers, suggesting that GAld is capable of forming brown carbon SOA.

## 1 Introduction

Glycolaldehyde (GAld) is a small, highly water-soluble molecule produced by the oxidation of many atmospheric precursors, including isoprene.^1^ Its global production rate from isoprene + ·OH oxidation has been estimated to be 42 Tg C year^–1^.^2^ In the gas phase, GAld reacts with photochemical oxidants to produce glyoxal and smaller species,^3,4^ with a midday lifetime of several hours.^1,5^ Like other small aldehydes, GAld is routinely detected in aqueous aerosols and cloudwater,^6−9^ even in remote areas, because of its ability to react with water to form a hydrate. The aqueous-phase photooxidation of GAld is a significant source of the aqueous secondary organic aerosol (aqSOA)^10^ because its reaction with dissolved OH radicals produces glyoxal, glycolic, oxalic, malonic, and succinic acids, larger oligomers,^2^ and organosulfate species.^11^ Additionally, GAld reacts in Maillard-type aqueous reactions with ammonium sulfate (AS)^12−14^ and amines (especially glycine)^13^ to form visible-light-absorbing products known as brown carbon (BrC) and also N-containing heterocyclic oligomers such as imidazoles and pyrazines.^14^ However, in laboratory studies where airborne droplets containing AS or amines were dried for several minutes with and without dissolved GAld, the presence of GAld slowed the evaporation process but did not measurably increase the size of the dried residual particles (except for 1:2 glycine/GAld mixtures).^15,16^ For this reason, it is not yet clear the extent to which Maillard dark reactions involving GAld can contribute to aqSOA or BrC production in atmospheric cloud droplets and aqueous aerosol.

In this study, we determine pH-dependent GAld + AS, GAld + glycine, and GAld + methylamine dark reaction kinetics using nuclear magnetic resonance (NMR) measurements of reactant loss rates in D_2_O solutions. Additionally, in large-chamber experiments we characterize SOA and BrC formation from GAld + glycine and GAld + AS + methylamine reactions as a function of relative humidity (RH). We identify sugar-like and N-containing aqueous-phase products formed after GAld uptake into aqueous aerosol particles containing AS, with and without cloud processing. We find that the exposure of GAld + AS aerosol to methylamine gas and dark and sunlit cloud processing increases the incorporation of both methylamine and ammonia into C_6_ and C_12_ GAld oligomers.

## 2 Materials and Methods

### 2.1 Chemicals and pH

All chemicals were purchased from Sigma-Aldrich unless otherwise stated. Stock solutions were made by the overnight hydrolysis of the GAld dimer, the dilution of 40% aqueous solutions of methylamine (Spectrum), or the dissolution of solid glycine or ammonium sulfate (AS), all in D_2_O (99.9%-D, Cambridge Isotopes) for NMR experiments or in 18 MΩ deionized water for chamber experiments. Amine or AS samples in D_2_O were pH-adjusted using acetic acid-*d*_6_ or sodium phosphate.

### 2.2 NMR Data Processing and the Derivation of Rate Constants

For reaction rate constant measurements, stock solutions in D_2_O with 1% v/v acetonitrile (an internal standard at 2.061 ppm) were vortex mixed in an NMR tube to reach an initial concentration of 0.5 M for each reactant at *t* = 0. The reaction mixture pH was measured immediately after mixing replicate samples in vials. Proton NMR spectra were recorded continuously (Varian, 400 or 500 MHz) over at least 16 h at room temperature. Table 1 lists the chemical shifts and locations of H atoms used for the NMR quantitation of reactant compounds. After phasing and baseline correction, integrated ^1^H peak areas were normalized by acetonitrile. GAld monomer NMR signals increased by ∼10% in the first hour after mixing because of an equilibrium shift from dimer to monomer in response to dilution by a factor of 2 caused by the mixing of the reactant solutions. Once GAld monomer–dimer equilibrium is achieved, declines in GAld NMR signals caused by other chemical reactions can be measured. Because GAld dimer hydrolysis has a half-life of ∼2 h,^17^ initial reaction rates were extracted from each NMR signal listed in Table 1 starting at *t* = 3 h. Normalized initial amine and aldehyde peak areas at *t* = 3 h were set equal to nominal concentrations of each reactant. Very slow reaction rates with rate constants of <2 × 10^–5^ M^–1^ s^–1^ could not be detected by NMR measurements of reactant loss.

**Table 1:** NMR Signals Used for the Quantification of Reactants

reactant molecule	functional group	NMR chemical shift (ppm)
hydrated glycolaldehyde monomer	CH_2_	3.50
hydrated glycolaldehyde monomer	CH	5.05
methylamine	CH_3_	2.58
glycine	CH_2_	3.55

The reaction order in this work was assumed to be first order in GAld and first order in amine or AS, like other Maillard reactions at low-to-moderate concentrations.^18−23^ Rate constants are given for active (aldehyde) forms of GAld rather than total GAld (aldehyde + hydrate forms). The second-order rate constant is derived from the measured initial reaction rate using the following equation^24^
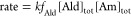
1where rate represents a measured initial reactant rate; *k* is the second-order rate constant in M^–1^ s^–1^; [Ald]_tot_ and [Am]_tot_ are the total concentrations in M of hydrated and unhydrated GAld and protonated and unprotonated amine (or ammonium), respectively; and *f*_Ald_ is the equilibrium fraction of GAld in aldehyde (not hydrate) form, determined to be *f*_Ald_ = 0.053 at room temperature by computational^17^ and NMR methods.^17,25^ We do not calculate pH-independent rate constants based on concentrations of deprotonated ammonia and amines because, as shown below, rates were not directly proportional to concentrations of the deprotonated species.

### 2.3 Chamber Experiments and ESI-HRMS Analysis

Cloud processing experiments took place in the 4.2 m^2^ CESAM chamber.^26,27^ Experimental start times were defined as the beginning of N_2_ and O_2_ addition to the evacuated chamber. Seed aerosol particles were generated from 1.8 mM AS, 2.0 mM glycine, or a mixture containing 1.8 mM AS and 50 mM GAld. A mixed AS + GAld aerosol was also collected directly on a filter without chamber exposure as a control. In three experiments in the chamber, seed aerosols were exposed to various combinations of gas-phase GAld, methylamine, one to two cloud events of 5–10 min duration each, and/or 60–75 min of simulated sunlight. Cloud events (supersaturation) were triggered by a combination of expansion-cooled water vapor injection and a gradual, 10% pressure reduction. The evolution of cloud droplet size distributions was characterized from a chamber flange by optical scattering (Palas Welas Digital 2000, 0.5 to 15 μm range).^26^ Experimental conditions are summarized in Table 2. Gas-phase species were monitored by PTR-MS (KORE Tech. Series II) and sensors for RH, NOx, NO, NO_2_, and ozone. PTR-MS signals for GAld at *m*/*z* 61 contain a significant contribution from the contaminant molecule acetic acid, which was detected whenever water vapor was added to the chamber. However, GAld and acetic acid contributions to *m*/*z* 61 signals were deconvoluted using *m*/*z* 43 because the two compounds have very different *m*/*z* 61/43 ratios, as shown in Figures S1 and S2.

**Table 2:** Summary of Chamber Experiments Involving Glycolaldehyde

expt	aerosol seed	[GAld]_g_ (ppm)	[MeAm]_g_ (ppm)	sun	clouds (no.)	filter collected	figures
1	glycine	1	0	no	1	no	3, S1, and S3
2	AS/GAld	0	1	yes	2	yes	4, S2, and S4
3^a^	AS/GAld	0	0	no	0	yes	
4	AS	0.3	1	yes	2	no	S5

a Control experiment: seed particles were collected directly on the filter without any exposure to methylamine gas, simulated sunlight, or cloud events in the chamber.

Aerosol physical and optical properties were monitored by scanning mobility particle sizing (SMPS, TSI), particle-into-liquid sampling (PILS)-waveguide UV–vis absorbance spectroscopy (1 m path length), and cavity-attenuated phase shift single-scattering albedo (CAPS-ssa, Aerodyne, 450 nm) spectroscopy.^28^ SMPS and CAPS-ssa data, both collected after drying aerosol through a Nafion sampling tube, were used to back-calculate time-dependent complex indices of refraction using an IDL routine over a 2-D range of *n* (1.33 to 2.00, step 0.01) and *k* values (0 to 0.050, step 0.001). Shape factors (1 to 1.1, step 0.02) were tested, but no evidence of nonsphericity (shape factors >1.00) was found. All *n* and *k* combinations that produced extinction and scattering values that matched observations within the measurement uncertainty were retained and then averaged to produce the reported *n* and *k* values. Because aerosol-phase total organic carbon was not quantified, PILS-waveguide data was converted to mass absorption coefficients (MAC) in this study only in experiments where aerosol growth was observed (expt. 2) or where seed particles contained glycine (expt. 1) because in these cases we could estimate the total organic carbon in the aerosol. TOC estimates and MAC calculations are described in the Supporting Information.

After chamber processing concluded, aerosol samples were collected at 15 L/min on Teflon filters over 16 h while the chamber pressure was held constant with a compensating dry N_2_ inlet flow. Chamber and control filters were frozen at −20 °C until extraction by acetonitrile immediately prior to ESI-HRMS analysis (Surveyor Plus system with an HPLC pump, autosampler, and PDA detector, an IonMAX electrospray ionization (ESI) source, and a high-resolution LTQ-Orbitrap mass spectrometer from Thermo Electron).^29^ The details of the ESI-HRMS experimental setup, data acquisition, peak deconvolution, and molecular formula assignment have been described previously.^30^ We report exact masses of all peaks detected with areas greater than 10^6^ and elevated relative to blank extract runs. No unusual safety hazards were encountered during the course of this work.

## 3 Results

### 3.1 Bulk Aqueous-Phase Glycolaldehyde Reaction Kinetics

**Figure 1:**
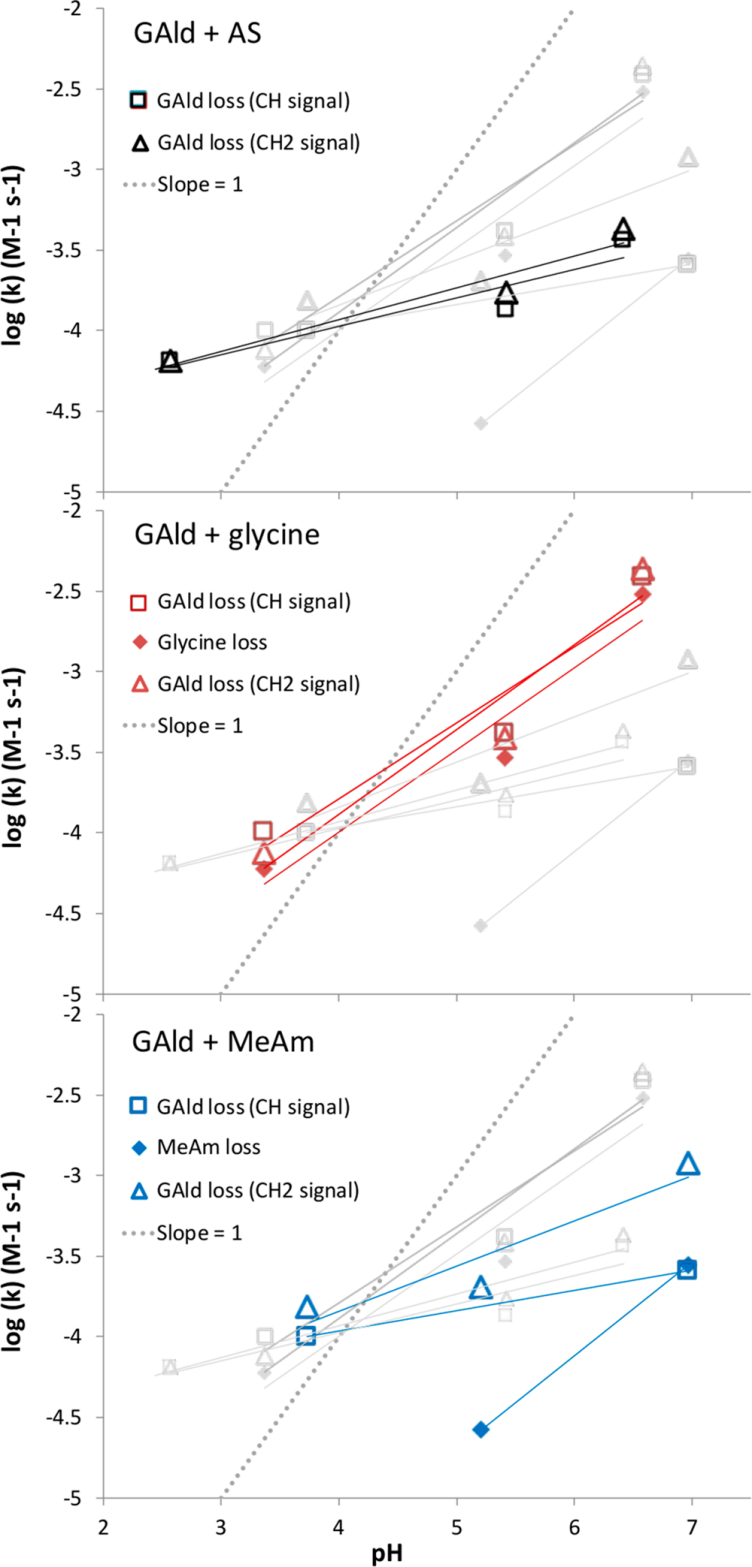
Apparent second-order rate constants (M^–1^ s^–1^) measured by ^1^H NMR for bulk aqueous phase glycolaldehyde reactions at room temperature. Identical data is shown in each panel, along with a slope = 1 dotted line for comparison. Individual panels highlight reactions with AS (black, top), glycine (red, middle), and methylamine (blue, bottom); initial concentrations = 0.5 M. Rate constants are calculated from losses of reactant signals: glycolaldehyde losses were followed with the CH_2_ signal (3.50 ppm, open triangles) and the CH signal (attached to the hydrated carbonyl, 5.05 ppm, open squares). Amine losses (filled diamonds) were calculated from methylamine CH_3_ (2.58 ppm) or glycine CH_2_ (3.55 ppm) signals. AS losses could not be measured, and methylamine losses at pH 3.7 were below the method detection limit.

A summary of second-order rate constants derived from NMR measurements of reactant loss rates in D_2_O is shown in Figure 1. These rate constants were calculated using total (protonated + unprotonated) concentrations of ammonia or amines present. Rate constants for all three reactions (glycolaldehyde + methylamine, glycine, or AS) were smaller than those measured for respective glyoxal or methylglyoxal reactions^24^ but showed a significant pH dependence, as expected for Maillard-type chemistry. If reaction rates were proportional to the concentrations of unprotonated ammonia or amine species, then the least-squares fits in Figure 1 would have slopes = 1 (shown as a gray dotted line in each panel for comparison). Instead, the pH dependence is substantially less than that (i.e., a slope of 1 is outside the ±3σ range of the least-squares fits). Furthermore, if reaction rates were solely a function of deprotonated nitrogen atom concentrations, then GAld + AS rate constants would be the highest at all pH values because ammonia is a weaker base than either amine species, so a greater fraction remains unprotonated. For GAld reactions with AS and glycine, the rates appear to depend more strongly on pH above pH ∼5. Below pH 5, the pH dependence appears to flatten, and GAld loss rates converge for all three reaction mixtures. Finally, in GAld + methylamine mixtures (blue symbols) with pH <7, loss rates of methylamine are substantially less than those of GAld.

These observations suggest that more than one reaction mechanism is in operation across the pH 2–7 range. At near-neutral pH, the reaction between reduced nitrogen and the carbonyl functional group appears to be the main sink for both compounds, causing a steeper pH dependence. At lower pH, an acid-catalyzed GAld self-reaction appears to be the dominant sink, consistent with the similar GAld loss rates observed at low pH in all three reaction mixtures. GAld aldol self-reactions are also catalyzed by ammonium and aminium ions,^20,23,31^ which may explain why methylamine loss rates are far less than GAld loss rates below pH 7. For the GAld + glycine reaction, the ratio of glycolaldehyde to glycine loss rates stayed constant across the pH range of 1.41 (±0.07) to 1, in contrast to the GAld + MeAm reaction. This indicates both a non-negligible role of GAld self-reactions even at neutral pH and some level of glycine reactivity even under acidic conditions. Interestingly, GAld + glycine mixtures have been shown to brown much more efficiently than GAld + MeAm or GAld + AS mixtures at pH 4.^15^ This difference must be due to the formation of products with higher molar absorptivities in the GAld + glycine system because the GAld loss rate kinetics are similar for all three systems at this pH.

**Figure 2:**
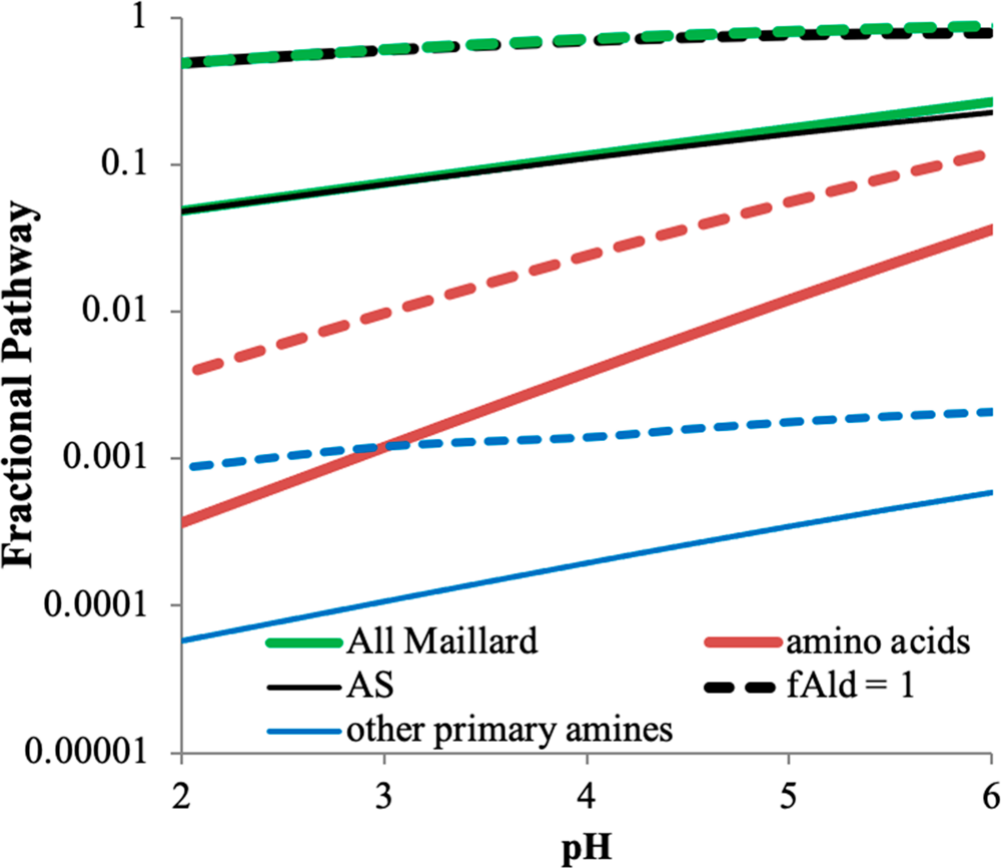
Estimation of the fraction of aqueous-phase GAld reacting via various Maillard pathways in daytime marine aerosol as a function of pH, calculated by assuming *f*_Ald_ = 0.053 (solid lines) or *f*_Ald_ = 1 (dashed lines, [NH_4_^+^] = 3 M; free amino acids = 0.1 M, all reacting with GAld at the rates measured here in bulk D_2_O for glycine; other primary amines = 4.3 × 10^–3^ M, all reacting with GAld at the rates measured in bulk D_2_O for methylamine; and [OH radical]_aq_ = 1 × 10^–13^ M). GAld + AS reaction (black lines), GAld + amino acid reactions (red lines), GAld + amine reactions (blue lines), and the sum of all Maillard pathways (green lines, dominated by the GAld + AS reaction) are shown.

By making an initial assumption that these pH-dependent rate constants measured for the loss of GAld CH_2_ groups in bulk liquid with glycine and methylamine are applicable to reactions with all amino acids and other primary amines, respectively, in suspended aqueous aerosol and cloud droplets, we can estimate the relative size of various atmospheric sinks for aqueous-phase glycolaldehyde. For this estimation, we used typical cloudwater concentrations of radical species ([OH radical] = 1 × 10^–13^ M),^32^ amine aqueous aerosol concentrations enriched by a factor of 10^4^ over measured concentrations in marine rain (resulting in free amino acids = 0.1 M, other primary amines = 4.3 × 10^–3^ M),^33^ and [NH_4_^+^] = 3 M, its equilibrium concentration in AS aerosol at 95% RH.^34^ (Some laboratory studies have used [NH_4_^+^] = 6.2 M,^35,36^ its equilibrium concentration at 90% RH.) GAld + OH radical reaction rates were calculated using *k*_OH_ = 1.5 × 10^9^ M^–1^ s^–1^.^2,37^ The results are summarized in Figure 2. If our rate constants measured here in bulk D_2_O are applied to aqueous aerosol particles, then Maillard-type reactions between glycolaldehyde and reduced nitrogen compounds would be less important than oxidation by dissolved OH radicals: we estimate that ∼20% of aqueous GAld would react at pH 5.5 by Maillard pathways during the day. However, if we set *f*_Ald_ = 1.0 instead of 0.053 to simulate the activation of GAld carbonyl groups at the air–water interface, ∼84% of aqueous GAld would react at pH 5.5 by Maillard pathways during the day, mostly by reacting with AS. Setting *f*_Ald_ = 1.0 instead of 0.053 results in a factor of ∼20 acceleration of reaction rates in aerosol particles relative to bulk liquid, which is quite modest compared to that observed for glyoxal–AS or glyoxal–amine reactions.^18,38^ Furthermore, fast photolytic radical-initiated oligomerization reactions between aldehyde and amine species in suspended aqueous aerosol particles have also been reported^39^ but are not included in these estimates. We explore GAld reactions in aqueous aerosol in the laboratory measurements described in the next section.

### 3.2 Glycolaldehyde Reactions in an Aerosol Cloud Chamber

To test whether GAld reactions with reduced nitrogen species can occur in suspended aqueous aerosol particles on a time scale of minutes to hours (rather than days), a short series of chamber experiments were performed (Table 2). In experiments 2 and 3, aerosol particles were collected on filters for LCMS analysis.

**Figure 3:**
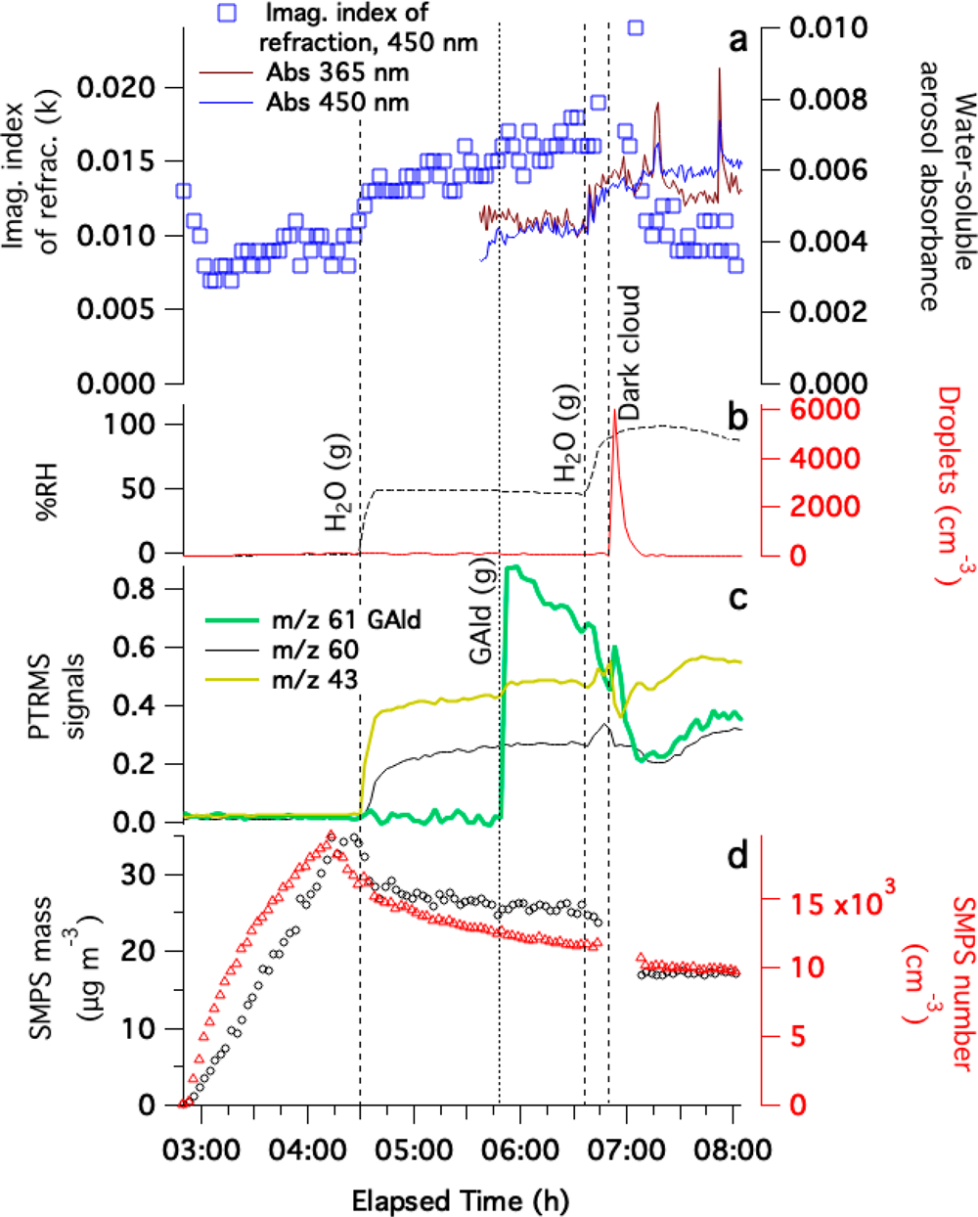
Summary of experiment 1, dried glycine seed particles exposed to GAld gas and cloud processing in the dark. (a) Time-dependent imaginary part of the index of refraction of dried aerosol extracted from CAPS-ssa data at 450 nm (blue squares) and the absorbance of water-soluble aerosol material sampled by PILS at 365 (brown line) and 450 nm (blue line). (b) Relative humidity (black dotted line) and cloud droplet counts (red line, right axis). (c) Dilution-corrected PTR-MS signals for *m*/*z* 43 (acetic acid fragment), *m*/*z* 60, and the GAld-attributed portion of *m*/*z* 61. (d) Dilution-corrected SMPS number density (red triangles, right axis) and mass (black circles). Additions of GAld gas (dots), water vapor addition, and cloud events (dashes) are labeled with vertical lines. For CAPS extinction, scattering, and albedo data, see Figure S3.

In experiment 1 (Figures 3 and S3), diffusion-dried glycine seed aerosol was exposed to water vapor, 1 ppm gas-phase GAld, and cloud processing in the dark. Increasing the chamber RH from dry to 50% caused an 18% loss of glycine seed dried particle mass, the introduction of a few trace contaminant gases along with the water vapor (such as acetic acid detected by PTR-MS at *m*/*z* 43 and 61), a large increase in the mass-based aerosol scattering efficiency, and a corresponding small increase in the imaginary part of the index of refraction at 450 nm from 0.009 to 0.013. Because 50% RH is well below the deliquescence point for glycine aerosol (∼95% RH),^40^ it appears that surface reorganization by adsorbed water is responsible for the observed changes in optical properties, which were measured after drying the aerosol. (The PILS-waveguide UV/vis absorbance baseline was unstable for the first 5.5 h of the experiment.) The addition of 1 ppm GAld gas (detected by PTR-MS at *m*/*z* 61) did not cause significant changes in the seed particle size or optical properties, indicating that GAld uptake into adsorbed water layers is insignificant. However, aerosol deliquescence, followed immediately by 15 min of dark cloud processing, resulted in the loss of 67% of GAld from the gas phase. (Much of this GAld may have gone to the walls; we cannot quantify particle growth during cloud processing because at least 30% of the aerosol mass was lost due to wet deposition.) Cloud processing also increased the absorbance observed by PILS in wet-sampled aerosol at 365 and 450 nm from 0.0042 to 0.0060 but reduced the imaginary part of the index of refraction at 450 nm in dried aerosol by a factor of 2. The loss of gas-phase GAld corresponding to increased absorbance in wet-sampled aerosol suggests that at least some GAld was taken up into the aerosol/droplet aqueous phase, where it reacted with glycine to reversibly form brown carbon on a 15 min time scale. However, this BrC was not stable against drying/evaporation. In the hour after the cloud event, 38% of the GAld taken up from the gas phase was slowly released back to the gas phase as the RH declined to 90%. While much of this release likely came from the chamber walls, some of it likely came from aerosol particles: the slow but nearly complete release of GAld from fully dried airborne droplets has been observed in an earlier study and held as evidence of reversible oligomer formation.^15^

**Figure 4:**
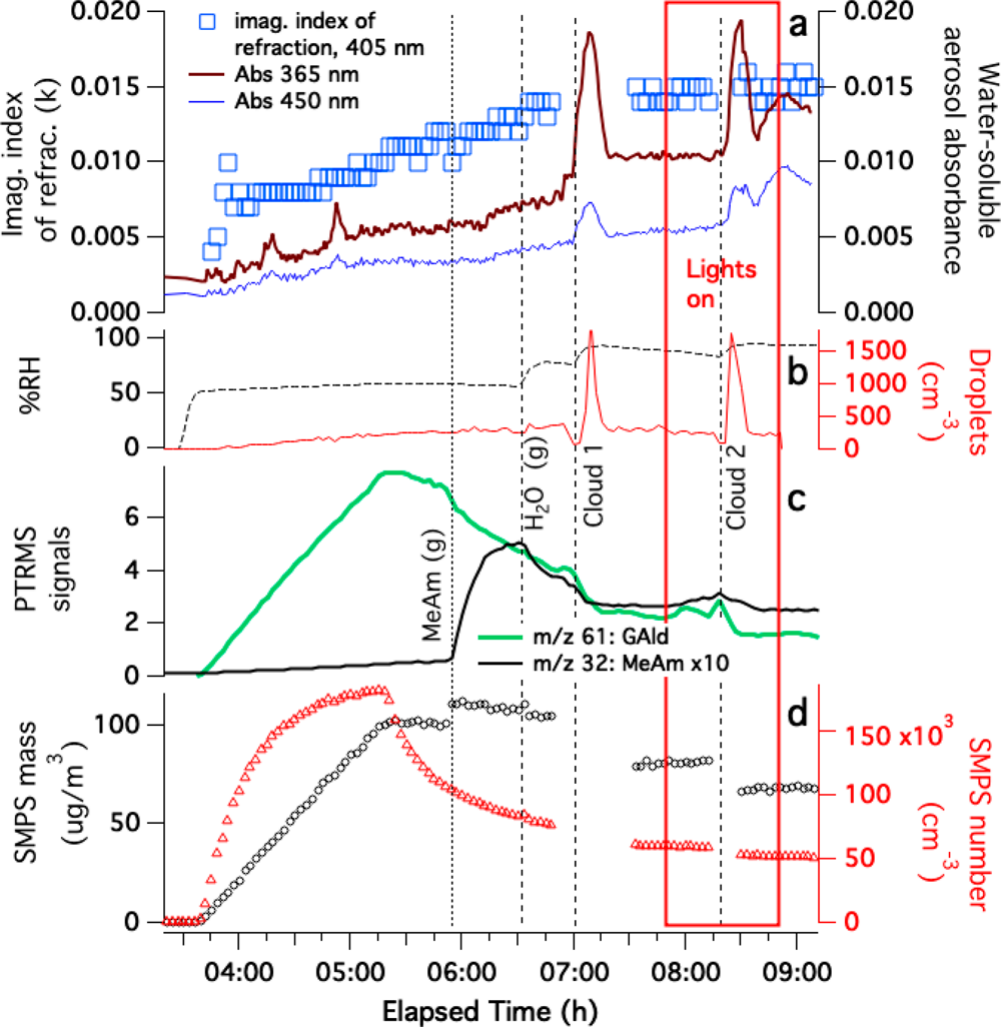
Summary of experiment 2, seed particles generated from 1.8 mM AS/50 mM GAld solution, added to a humidified chamber without drying and then exposed to methylamine gas, cloud processing, and simulated sunlight. (a) Time-dependent imaginary part of the index of refraction of dried aerosol extracted from CAPS-ssa data at 450 nm (blue squares, left axis) and the absorbance of water-soluble aerosol material sampled by PILS at 365 (brown line) and 450 nm (blue line). (b) Relative humidity and cloud droplet counts. (c) Dilution-corrected PTR-MS signals for methylamine (black line, *m*/*z* 32 signals multiplied by 10) and GAld-attributed *m*/*z* 61 signals (green line). (d) Dilution-corrected SMPS number density and mass. For CAPS extinction, scattering, and albedo data, see Figure S4.

Experiments 2 and 3 involved mixed AS + GAld seed particles that were either collected without chamber exposure as a control (experiment 3) or exposed to 1 ppm methylamine gas, dark cloud processing, and cloud processing in 60 min of simulated sunlight before filter collection (experiment 2). Reaction product ions detected in filter extracts by ESI-HRMS in the two experiments are summarized in the next section; optical and physical parameters measured during chamber exposure (experiment 2) are summarized in Figure 4. Seed particles were added without diffusion drying to the chamber at 50–58% RH; under these conditions, aerosol droplets containing AS cannot effloresce but remain aqueous-phase particles. PTR-MS signals at *m*/*z* 61 indicate that a substantial amount of GAld evaporated from the seed particles (and likely also from the liquid used in the atomization process), reaching a peak of 8.7 ppm in the chamber at the end of the seed particle addition. Even before any further additions to the chamber, absorbance measured in PILS-sampled aerosol reached 0.0055 at 365 nm and 0.0032 at 450 nm, presumably because of brown carbon formed by GAld + AS reactions in the aqueous aerosol particles.

The addition of 1 ppm methylamine gas to the chamber caused an immediate 9% increase in the dried aerosol mass, accelerated the loss rate of GAld from the gas phase (some of which may have gone to the chamber walls), and triggered the start of an upward trend in MAC_365 nm_. These observations suggest that the reactive uptake of both methylamine and GAld into the deliquesced aerosol particles occurred, followed by measurable BrC formation. Subsequent dark cloud and sunlit cloud events caused the PILS-sampled aerosol absorbance to spike while irreversibly drawing down ∼40% of gas-phase GAld in the chamber, suggesting rapid BrC formation involving GAld. Although aerosol absorbance declines as each cloud dissipates, the absorbance after each cloud remains 30–40% higher than it was before the cloud. Unlike in experiment 1, cloud processing (even in simulated sunlight) did not reduce the imaginary part of the index of refraction measured in dried aerosol at 450 nm. These observations indicate that BrC produced in multiphase GAld + AS + methylamine reactions is resistant to drying and also to hydrolysis and photobleaching, as was recently observed in a study conducted on bulk liquid water solutions.^39^

### 3.3 Aerosol-Phase Reaction Products

**Scheme 1:**
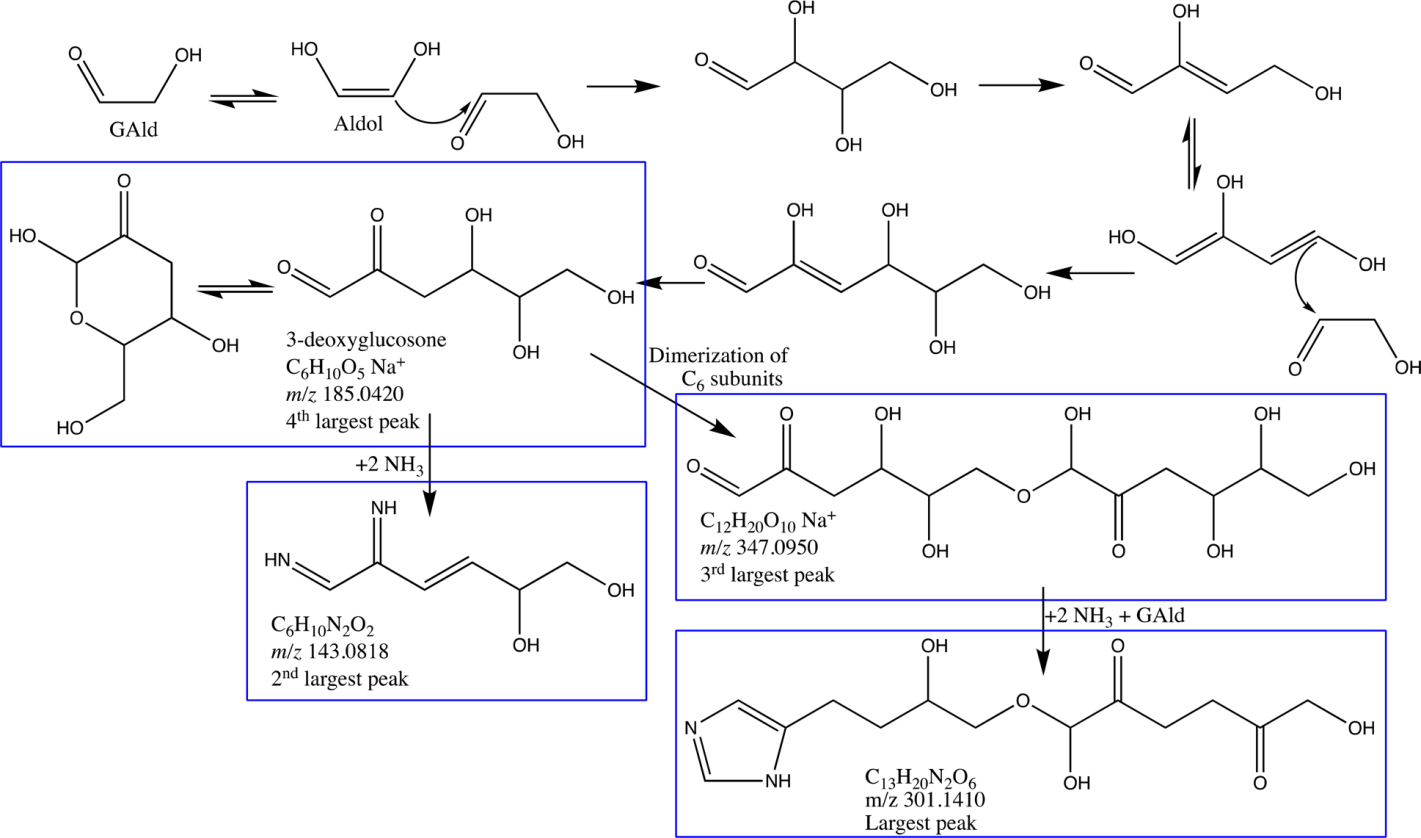
Proposed Dark Mechanism for the Preferential Formation of C_6_ and C_12_ Glycolaldehyde Oligomers and Their Reactions with Ammonia in Aqueous Aerosol Particles Blue boxes designate the four largest peaks detected in the unprocessed GAld + AS seed aerosol control (experiment 3)

Aerosol-phase products detected by LC-ESI-HRMS in GAld + AS seed aerosol extracts before (experiment 3) and after exposure to methylamine gas, and both dark and sunlit cloud processing (experiment 2) are shown in Table 3. The four largest peaks detected in the GAld + AS seed aerosol (without chamber exposure) are listed in Table 3. Aerosol-phase GAld oligomers are observed, some of which contain nitrogen, indicating that some fraction of GAld reacted with itself or with AS rather than evaporating into the gas phase. We note that that 99.8% of the peak area in the chromatogram of unprocessed GAld + AS aerosol was attributed to molecules built from GAld trimer units (C_6_, C_12_, and C_24_). Like methylglyoxal, GAld can oligomerize via aldol condensation or acetal formation, forming products by either pathway with identical formulas but different linkages and structures. Although any number of GAld units could oligomerize via acetal formation, we propose that aldol condensation preferentially generates the C_6_ intermediate 3-deoxyglucosone (*m*/*z* 185.0420), which is in equilibrium with a stable cyclic form (Scheme 1), allowing it to accumulate without further aldol additions of GAld monomers. Two 3-deoxyglucosone units can then link via an acetal reaction to form a C_12_ oligomer, bypassing C_8_ and C_10_ forms, analogous to sugar chemistry. Thus, the observed preference for products built from C_6_ and C_12_ units suggests that GAld, like methylglyoxal,^35^ forms oligomers primarily by aldol condensation in aqueous aerosol particles.

**Table 3:**
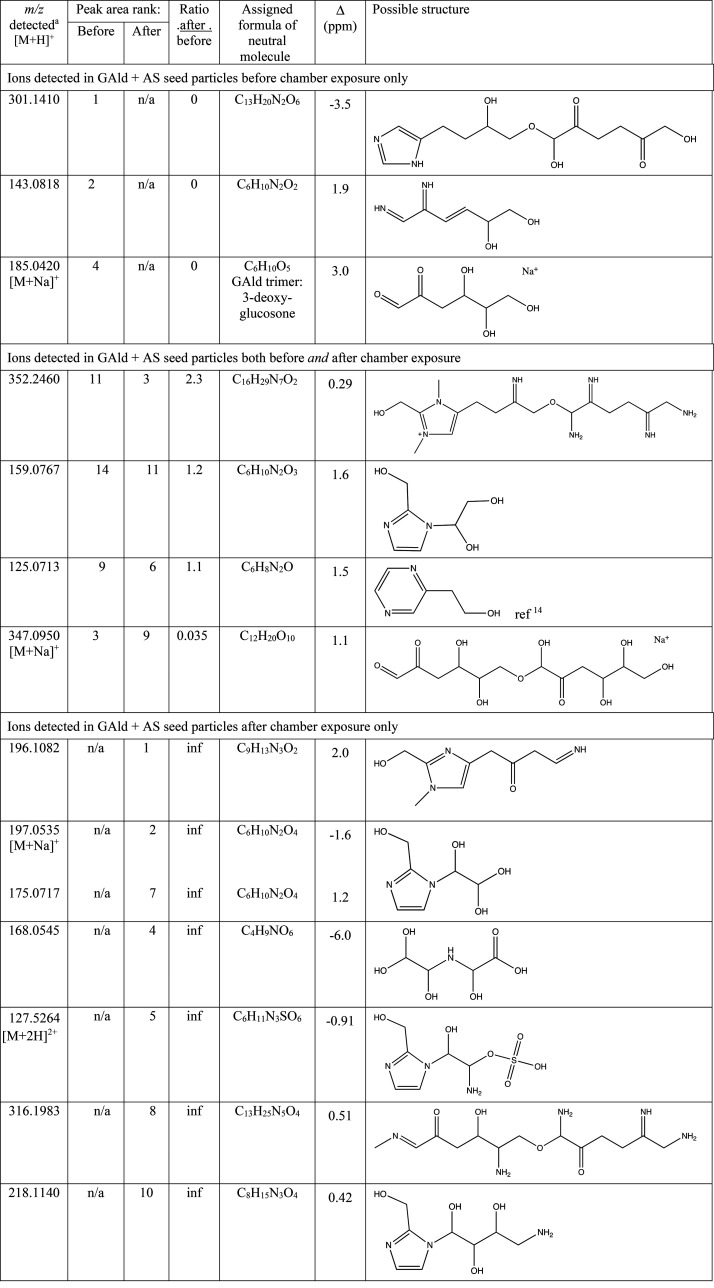
Aerosol-Phase Reaction Products Detected in Glycolaldehyde + AS Seed Particles before and after Exposure to Methylamine Gas, Cloud Processing, and Simulated Sunlight^a^

a Major peaks were detected in GAld + AS aerosol by positive ion mode ESI-MS before (experiment 3) and after (experiment 2) chamber exposure to methylamine gas, cloud processing, and simulated sunlight. a: [M + H]^+^ ion, unless otherwise stated. n/a: peak not detected in the given experiment. inf: The “after/before” ratio is infinitely large (division by zero).

Both 3-deoxyglucosone and its C_12_ “disaccharide” can then be converted by dark reactions with ammonia to diimines and imidazoles, resulting in the two largest peaks detected in unprocessed GAld + AS seed particles: a proposed C_6_H_10_N_2_O_2_ diimine species (*m*/*z* 143.0818) and a C_13_H_20_N_2_O_6_ imidazole derivative (*m*/*z* 301.1410, 100-fold larger peak). Imidazole derivatives are typical thermodynamic end points for Maillard dark chemistry.^41^ In unprocessed GAld + AS seed aerosol (experiment 3), the average number of N atoms/molecule (weighted by peak area)^39^ was 2.0, which supports the dominance of diimine and especially imidazole products in terms of concentrations, electrospray ionization efficiencies, or both.

After exposing the GAld + AS seed aerosol to methylamine, cloud processing, and simulated sunlight (experiment 2), the average number of N atoms/molecule detected increased from 2.0 to 2.9. Detected exact masses, proposed formulas, and possible molecular structures are shown in Table 3. Approximately half of this increase in N atoms was due to methylamine incorporation in detected products. The incorporation of ammonia also increased, likely because of the exchange reaction of methylamine with ammonia in the AS-containing aerosol increasing the concentration of dissolved, unprotonated ammonia. The uptake of methylamine into the aerosol particles, seen as an increase in aerosol mass in Figure 4, could also increase the concentration of unprotonated ammonia by raising the pH of the aqueous aerosol phase.

Methylamine exposure, cloud processing, and simulated sunlight reduced the detected abundance of GAld oligomers that do not contain N atoms by 98% and appeared to increase the diversity of product types. N-Derivatized C_6_ and C_12_ GAld oligomer products are still dominant (making up 68% of the product molecules, weighted by peak area), but a proposed organosulfate imidazole product (*m*/*z* 127.5264) was also generated during chamber exposure, and the concentration of a pyrazine product identified by Grace et al.^14^ (*m*/*z* 125.0713) increased slightly. Four imidazole derivatives formed by the nucleophilic attack of the imidazole:NH group on GAld monomers or dimers (analogous to the formation of hydrated *N*-glyoxal-substituted 1*H*-imidazole in the glyoxal + AS system)^21,22^ are also detected in chamber-exposed GAld + AS aerosol. The average number of conjugated double bonds per detected molecule did not increase in the chamber-exposed aerosol compared to in the seed particles, indicating that the increased MAC_365_ observed in PILS-sampled aerosol after cloud processing in the chamber is likely due to other factors. These factors include the greater nitrogen incorporation in organic product molecules (2.9 vs 2.0 N/molecule detected) and the additional derivatization of imidazoles observed in the chamber-processed aerosol.

## 4 Discussion and Conclusions

### 4.1 Bulk vs Aerosol Reaction Rates

Aerosol particles in experiments 2–4 were never dried until they were collected on filters; the pH of the deliquesced AS aerosol has been estimated to be 3.2, with gas-phase ammonia levels in the ambient range having little effect on the pH.^42^ At this pH, our rate measurements suggest that GAld + AS and GAld + methylamine reactions have similar rate constants (*k* ≈ 10^–4^ M^–1^ s^–1^). Thermodynamic modeling (E-AIM (iii)^34^ indicates that in the aqueous AS aerosol at 58% RH, ammonium and sulfate ions have a combined mole fraction of 0.4, with ammonium concentrations of ∼25 molality (*m*). Upon methylamine uptake, some ammonium will be exchanged with methylaminium ions, but the total concentration of ammonium and methylaminium ions is likely to remain very high. If the aerosol pH remains at 3.2 after methylamine uptake, then the lifetime of any dissolved GAld with respect to amine or ammonia reactions will be ∼2 h. If, however, GAld’s dihydrate/aldehyde equilibrium shifts strongly toward the aldehyde at the air–water interface (for *f*_Ald_ = 0.053 to 1), as has been suggested for other small aldehydes,^24^ then the lifetime of GAld could become as short as ∼7 min. This time scale of minutes is more consistent with our aerosol observations: the retention of some GAld in evaporating GAld + AS aerosol droplets (at 58% RH), the detection of GAld oligomers in these particles, and the accelerated uptake of GAld from the gas phase upon introduction of methylamine gas into the chamber (also at 58% RH), all of which took place in minutes.

In contrast, in a more dilute, postcloud environment at 98% RH, where [NH_4_^+^] = 1.1 *m*, the predicted lifetimes for nonhydrated (aldehyde-form) GAld molecules with respect to AS/amine reactions would lengthen to ∼2.5 h and lifetimes in large, activated cloud droplets would be even longer. Under these high-RH conditions, bulk-phase reaction kinetics, even with a 20-fold acceleration due to surface equilibrium shifts to aldehyde-form GAld (for *f*_Ald_ = 0.053 to 1), are still an order of magnitude slower than the spikes in absorbance or the irreversible losses of GAld(g) observed during each 10 min cloud event. A possible explanation is further surface effects, where GAld preferentially partitions to and reacts at an air–water interface crowded with other surface-active species. Several other aldehyde + AS/amine reaction systems generate surface-active species,^35,43,44^ including during photolysis.^45^

### 4.2 Atmospheric Significance

In the atmosphere at moderate RH, the presence of other substances in aqueous aerosol particles would likely lower effective reactant concentrations,^46^ and organic species could lose access to ammonium and aminium salts by “salting out” or by the liquid–liquid phase separation of organic and aqueous phases.^47^ Both factors would slow down reactions among GAld, AS, and amine species in aqueous aerosol at moderate humidity levels. However, at RH near 100%, Henry’s law (rather than other dissolved species) would control GAld concentrations in the droplet, and surface activity could cause the surface concentrations of GAld or first-generation GAld + AS/amine products to reach high levels, even though typical atmospheric GAld gas concentrations are significantly lower than the 0.3–1 ppm concentrations used in experiments 1 and 4. Additionally, cross reactions between GAld and other aldehydes as they react with AS and amine species, especially those that generate additional surface-active intermediates, may accelerate the incorporation of GAld into BrC oligomers. Cloud processing of GAld in the presence of AS and amine species can therefore be expected to produce brown carbon under atmospheric conditions.

## Supporting Information Available

Description of PTR-MS signal corrections for changing water vapor levels; figures representing the deconvolution of acetic acid and GAld signals at *m*/*z* 61; figures summarizing chamber experiments 1, 2, and 4 including extinction, scattering, and single-scattering albedo measurements at 450 nm and a listing of all peaks detected by ESI-HRMS in the aerosol extracts. The raw data from aerosol–cloud chamber experiments is available in a data repository.^48^ (PDF)
