# Supplementary material for: Kinetics, Products, and Brown Carbon Formation by Aqueous-Phase Reactions of Glycolaldehyde with Atmospheric Amines and Ammonium Sulfate
Source: J Phys Chem A. 2022 Aug 4;126(32):5375–85. doi: 10.1021/acs.jpca.2c02606 (PMC9393862; doi:10.1021/acs.jpca.2c02606)
Supplement: Supplementary file 1 — jp2c02606_si_001.pdf [file jp2c02606_si_001.pdf]

Kinetics, Products, and Brown Carbon Formation by Aqueous-Phase Reactions of Glycolaldehyde with Atmospheric Amines and Ammonium Sulfate: Supplemental Information

*Alyssa A. Rodriguez,[1] Michael A. Rafla,[1] Hannah G. Welsh,[2] Elyse A. Pennington,[2] Jason R. Casar,[2] Lelia N. Hawkins,[2] Natalie G. Jimenez,[1] Alexia de Loera,[1] Devoun R. Stewart,[1†] Antonio Rojas,[1] Matthew-Khoa Tran,[1] Peng Lin,[3††] Alexander Laskin,[3] Paola Formenti,[4] Mathieu Cazaunau,[4] Edouard Pangui,[4] Jean-François Doussin,[4] David O. De Haan[1]**

[1] Department of Chemistry and Biochemistry, University of San Diego, 5998 Alcala Park, San Diego CA 92110 USA

[2] Department of Chemistry, Harvey Mudd College, 301 Platt Blvd, Claremont CA 91711 USA

[3] Environmental Molecular Sciences Laboratory, Pacific Northwest National Laboratory, Richland, Washington 99352, USA, and Department of Chemistry, Purdue University, West Lafayette IN 47907 USA

[4] Laboratoire Interuniversitaire des Systèmes Atmosphériques (LISA), UMR7583, CNRS, Université Paris-Est Créteil (UPEC) et Université de Paris, Institut Pierre Simon Laplace (IPSL), 94000 Créteil, France

†: now at Department of Chemistry, Sacramento City College, 3835 Freeport Blvd. Sacramento, CA 95822 USA

††: now at California Air Resources Board, 4001 Iowa Ave., Riverside, CA, 92507 USA

* Corresponding author contact: 011-1-619-260-6882, -011-1-619-260-2211 fax, ddehaan@sandiego.edu

Figures S1-S5, Table S1

10 pages

PTR-MS data was corrected for water signals, since the amount of water vapor present in the inlet sample flow affects the ionization of all other species. The equation for the water-corrected signal of a particular *m/z* ion (M_{cor}) at time *t* is:

$$M_{cor} = M_{raw} / (M_{39} + M_{21})$$

Where M_{raw} is the uncorrected signal for that ion at time *t*, M_{39} is the uncorrected signal for the $H_2O.H_2{}^{18}O.H^+$ water cluster at time *t*, and M_{21} is the uncorrected signal for the $H_2{}^{18}O.H^+$ hydronium ion at time *t*.

For experiments 1 and 2, total organic carbon (TOC) concentrations in chamber aerosol particles were estimated using seed particle compositions and time-dependent aerosol mass concentrations measured by scanning mobility particle sizing (SMPS). For glycine seed particles (experiment 1, where growth was not observed), TOC equals the aerosol mass concentration at a given time multiplied by the mass ratio of carbon to glycine (24 amu / 75 amu). For AS / GAld seed particles (experiment 2), AS was assumed to be non-volatile while 90% of GAld was assumed to evaporate, giving a seed particle that is initially 56% GAld by mass, or 56% × (24 amu / 60 amu) = 22% C by mass using the mass ratio of carbon to GAld. When aerosol mass declined due to wall losses, calculated TOC values were scaled proportionally. When aerosol mass increased (experiment 1), it was assumed that increase was due to methylamine uptake, so the SMPS-measured mass increase was multiplied by the mass ratio of carbon to methylamine (12 amu / 31 amu). Note that the mass ratio of carbon to GAld is almost identical to that of methylamine, so the TOC increase is not sensitive to the amount of GAld that also may have been taken up by aerosol particles. Using these methods provided a rough estimate of TOC values with ±50% uncertainties at time points late in experiments 1 and 2 when BrC formation was observed. Mass absorption coefficients (MACs)

were then calculated from absorbance values measured by the PILS – waveguide UV/vis spectrometer using the equation

$$MAC = \frac{2.303\,Abs}{b\,C}$$

where b is the pathlength (94 cm) and C is the TOC concentration estimated as described above.

The PILS – waveguide UV/vis spectrometer was a single-beam instrument recording absorbance from 200 – 800 nm relative to a reference spectrum of PILS outflow deionized water collected at the start of each experiment. In experiments where baseline instability was observed after the chamber experiment began (experiment 1), the instrument was disconnected from the chamber to collect a new reference spectrum. (This is the reason for the data gap before 5.5 h in the "water-soluble aerosol absorbance" data in experiment 1, as shown in Figure 3. Unfortunately, baseline instability continued for ~40 minutes even after the new reference spectrum was collected, as shown in the full dataset in Figure S3, ending only at 5.5 h.) Absorbance data collected at 450 nm was selected for the figures for comparison with data from the CAPS-ssa spectrometer, which measured aerosol extinction and scattering at 450 nm only, and this data was used to calculate the complex index of refraction at 450 nm. Absorbance data at 365 nm was also selected because absorbance signal-to-noise ratios were maximized in the PILS – waveguide UV/vis spectrometer at near this wavelength. Since brown carbon absorbance spectra are typically featureless, as also observed in this study at wavelengths >290 nm, absorbance trends tend to be similar at 365 and 450 nm, with slightly larger absorbance expected at 365 nm.

Experiment 4 (Figure S5) involved dry AS seed particles exposed to 0.3 ppm GAld and 1.0 ppm methylamine gases in the dry chamber, followed by dark cloud processing and then cloud processing in simulated sunlight. While GAld was lost very quickly from the gas phase after its

introduction to the dry chamber (PTR-MS *m/z* 61 signal), most of this loss must have been to the chamber walls, since particle growth was not observed by SMPS. In any case, the lack of growth observed for solid, dry aerosol particles when exposed to gas-phase GAld is consistent with experiment 1. Net growth was also not observed upon exposure of dry AS seed to MeAm gas. Humidification of the chamber to cloud conditions caused a 65% drop in PTR-MS methylamine gas signals (*m/z* 32) to reach a steady state. Each cloud event temporarily released 20 – 50% of previously-lost GAld back to the gas phase, likely by hydrolysis from the chamber walls.

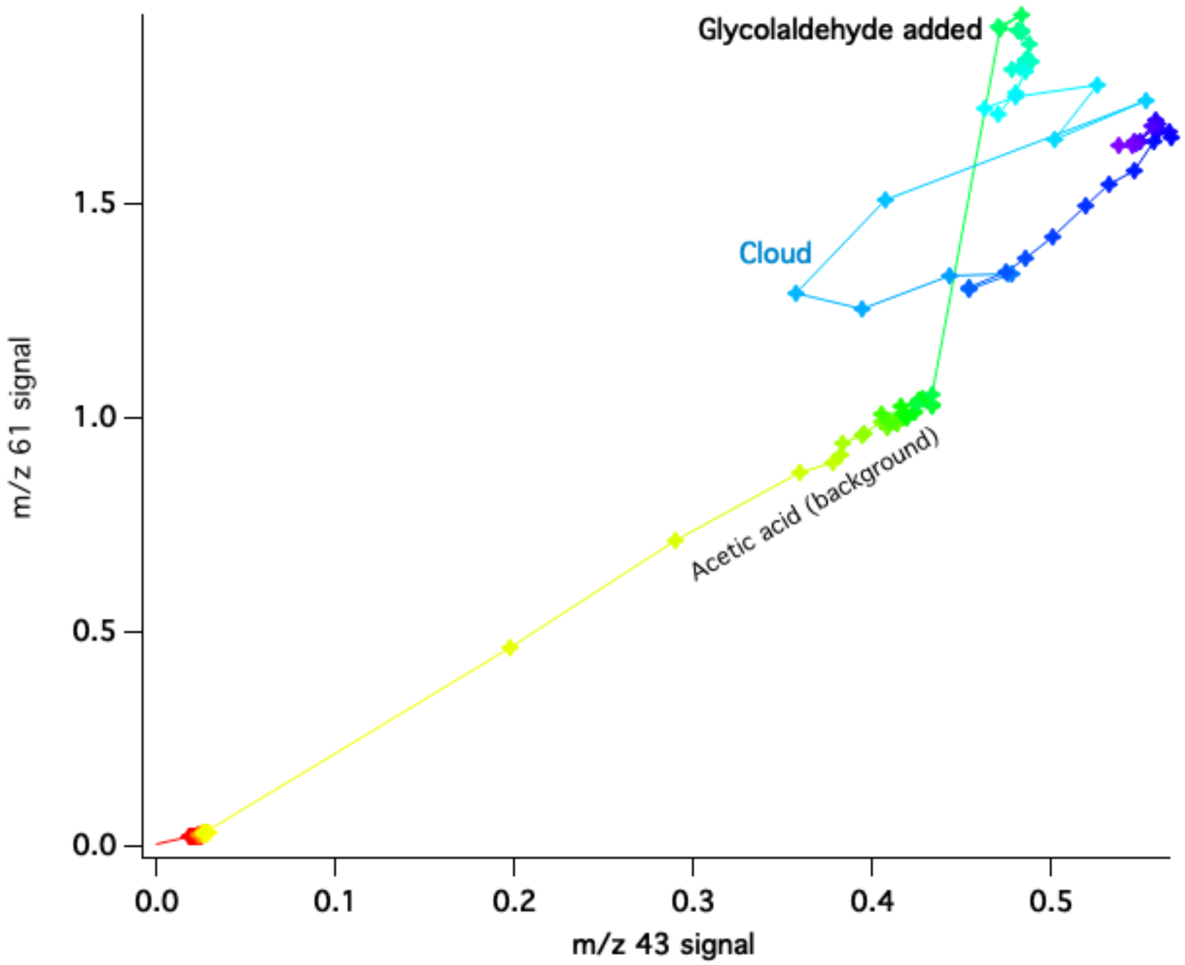

Figure S1: Basis of deconvolution of acetic acid and glycolaldehyde PTR-MS signals at *m/z* 61 using acetic acid fragment at *m/z* 43 (experiment 1). Acetic acid appeared when water was added to the chamber, then gas-phase glycolaldehyde was added. For each compound, *m/z* 61 / 43 ion signal ratios can be extracted from the slopes of the data upon each respective addition: slope = 2.51 for acetic acid and 23.3 for glycolaldehyde addition.

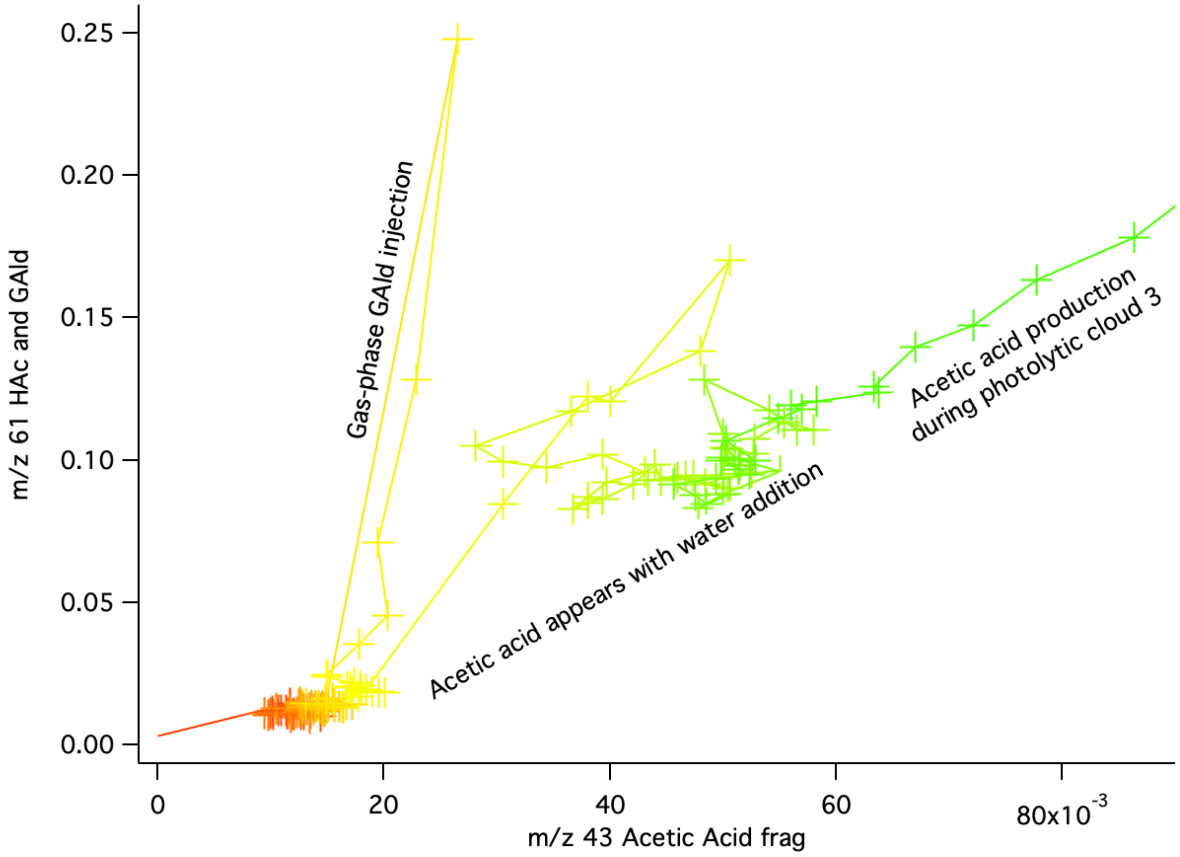

Figure S2: Deconvolution of acetic acid and glycolaldehyde PTR-MS signals at *m*/*z* 61 using ratio of signal with acetic acid fragment at *m*/*z* 43, experiment 2. Acetic acid appears when water is added to the chamber. For each compound, *m*/*z* 61 / 43 ion signal ratios can be extracted from the slopes of the data upon each respective addition: slope = ~1.9 for acetic acid and 19.1 for glycolaldehyde addition. The larger changes observed in experiment 1, and the order of addition (water then GAld) result in more accurate determination of slopes; therefore, slopes from experiment 1 were used to deconvolute *m*/*z* 61 signals in all experiments.

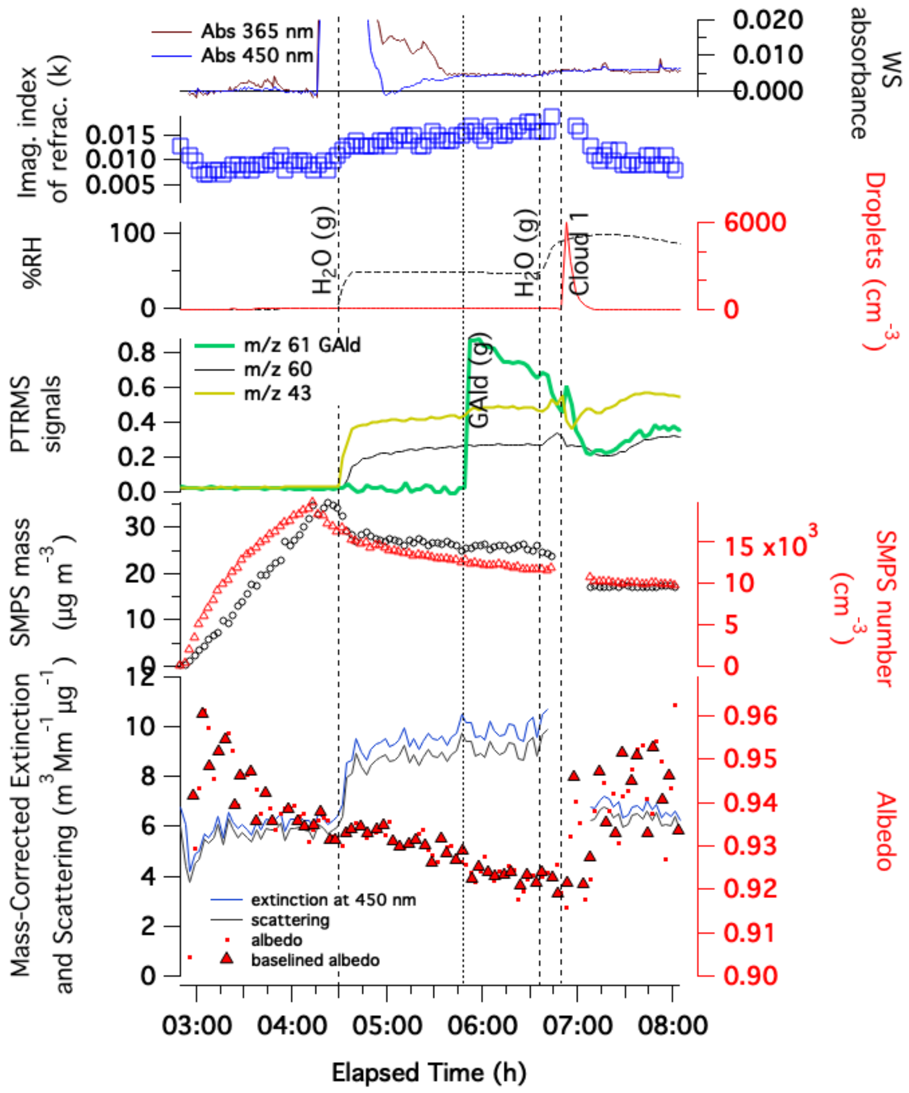

Figure S3: Summary of experiment 1, dried glycine seed particles exposed to GAld gas and cloud processing in the dark. Top panels: absorbance of water-soluble aerosol material sampled by PILS at 365 (brown line) and 450 nm (blue line) including unstable baseline before 5.5 h, and time-dependent imaginary part of the index of refraction of dried aerosol extracted from CAPS-ssa data at 450 nm (blue squares). Middle panels: relative humidity (black dotted line), and cloud droplet counts (red line, right axis), and dilution-corrected PTR-MS signals for *m/z* 43 (acetic acid fragment), 60, and GAld-attributed portion of *m/z* 61. 5th panel: dilution-corrected SMPS number density (red triangles, right axis) and mass (black circles). Bottom panel: extinction (blue line), scattering (black line), and albedo values (filled triangles measured immediately after instrument filter baseline, red dots measured at other times) measured by CAPS at 450 nm, 3-min averages. Additions of GAld gas (dots), water vapor and cloud events (dashes) are labelled with vertical lines.

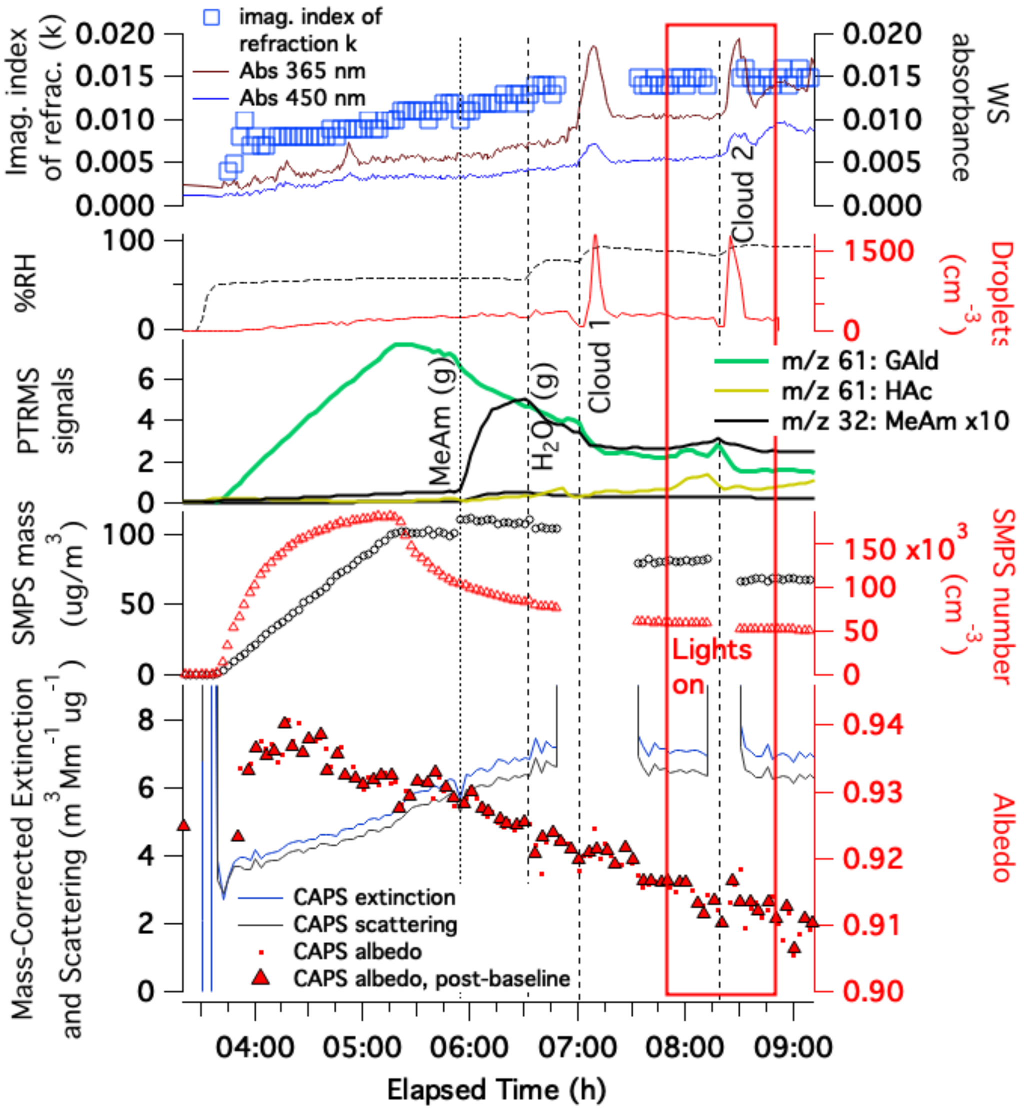

Figure S4: Summary of experiment 2, seed particles generated from 1.8 mM AS / 50 mM GAld solution, added to humidified chamber without drying, and then exposed to methylamine gas, cloud processing, and simulated sunlight. Time-dependent imaginary part of the index of refraction at 450 nm extracted from available CAPS-ssa data, absorbance of water-soluble aerosol material sampled by PILS, relative humidity, and cloud droplet counts (top 2 panels), dilution-corrected PTR-MS signals for *m/z* 32 (methylamine) and GAld-attributed (green) and acetic acid attributed (gold) portions of *m/z* 61 (middle panel), dilution-corrected SMPS number density and mass, and extinction, scattering, and albedo values measured by CAPS at 450 nm (bottom two panels).

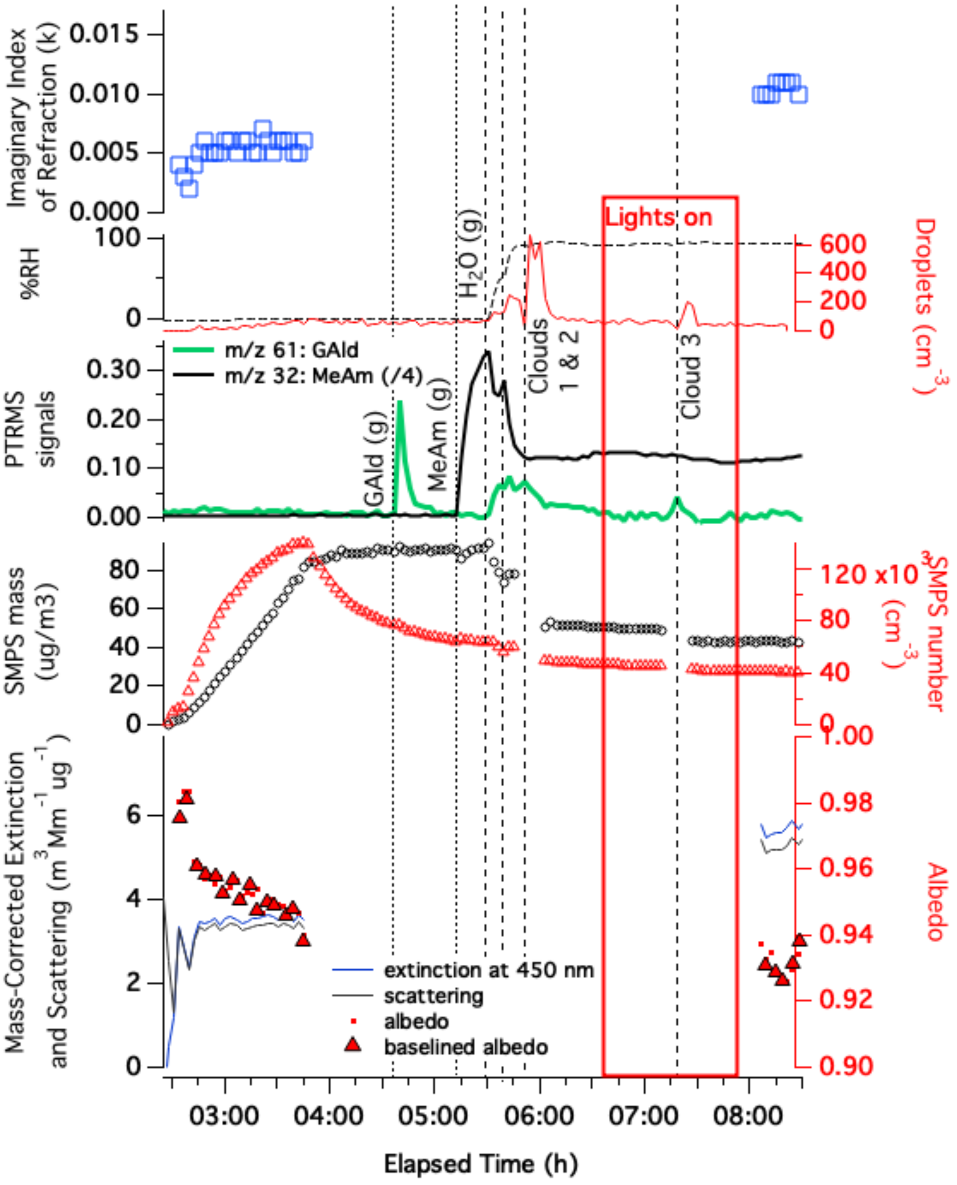

Figure S5: Summary of experiment 4, dried AS seed particles exposed to gas-phase GAld, MeAm, cloud processing, and simulated sunlight. Top panels: Time-dependent imaginary part of the index of refraction at 450 nm extracted from available CAPS-ssa data; relative humidity, and cloud droplet counts. Middle: dilution-corrected PTR-MS signals for *m*/*z* 32 (methylamine) and GAld-attributed portion of *m*/*z* 61 (middle panel). Bottom panels: dilution-corrected SMPS number density and mass, and extinction, scattering, and albedo values measured by CAPS at 450 nm. No CAPS data was collected between 4 – 8 h. No PILS / waveguide UV-vis data is available for this experiment.

Table S1: List of all ESI-HRMS peaks detected in aerosol extracts

Detected *m/z*	Proposed Formula	Delta mass (ppm)	Peak areas, Expt 3<br>Control	Peak areas, Expt 2<br>Chamber Exposed
301.141006	C13H20N2O6	-3.47	4.60E+07	
143.0817837	C6H11N2O2	1.88	2.29E+05	
347.0950425	C12H20O10 Na+	1.09	1.53E+05	5.34E+03
185.0420481	C6H10O5 Na+	2.96	6.66E+04	
202.1801659	C11H23NO2	2.64	6.17E+04	
329.0843402	C12H18O9 Na+	1.56	4.53E+04	
263.055528	C11H12O6 Na+	-9	2.59E+04	
399.2831212	C24H40O3 Na+	-11	9.44E+03	
125.0712994	C6H9N2O+	1.51	6.34E+03	6.76E+03
217.0499841	C10H10O4 Na+	-10.6	5.27E+03	
352.2459962	C16H30N7O2+	0.29	5.11E+03	1.16E+04
258.1926714	C11H23N5O2	1.27	4.93E+03	
154.0977554	C7H11N3O	1.83	3.85E+03	
159.076712	C6H10N2O3	1.61	3.40E+03	4.03E+03
215.1504106	C9H18N4O2	1.81	2.84E+03	
167.0467046	C10H8O Na+	3.44	2.05E+03	
196.1082204	C9H13N3O2	1.95		1.73E+04
197.0535484	C6H10N2O4 Na+	-1.65		1.36E+04
168.0545058	C4H9NO6	-6.03		1.10E+04
127.5263708	C6H13N3SO6 2+	-0.91		6.86E+03
175.0716701	C6H10N2O4	1.22		6.74E+03
316.1983193	C13H25N5O4	0.51		5.58E+03
218.1139909	C8H15N3O4	0.42		4.20E+03
Peak area sums			4.66E+07	9.31E+04
Number of peaks detected			16	11

Notes: Proposed formula is for neutral molecule, except for detected Na+ adducts, which have Na+ included in the formula. Delta mass (ppm) = ((calculated exact mass – measured exact mass) / calculated exact mass) x 10^6. Control experiment 3 is unprocessed GAld + AS seed particles. Experiment 2 is same type of seed particles after exposure to methylamine gas, cloud processing, and simulated sunlight in the CESAM chamber.
